# A Comprehensive Review on Virtual Articulators

**DOI:** 10.7759/cureus.52554

**Published:** 2024-01-19

**Authors:** Khushbu N Doshi, Seema Sathe, Surekha A Dubey, Anjali Bhoyar, Mithilesh Dhamande, Tanvi Jaiswal

**Affiliations:** 1 Prosthodontics, Crown and Bridge, Sharad Pawar Dental College and Hospital, Datta Meghe Institute of Higher Education and Research, Wardha, IND

**Keywords:** augmented reality, digital technology, digitisation, virtual reality, virtual articulator

## Abstract

The virtual articulator (VA) is a technology that simulates the jaw relation in a computer-generated setting. Augmented and virtual reality have been utilized as digital technology, which aids in many areas of dentistry and dental education. Today, a practicing dentist must keep up with the newer technologies, but with technology evolving so quickly it becomes challenging. In dentistry, the possibilities for digitization and technological advancements are limitless. Virtual articulators (VAs) allow a complete occlusion analysis using dental models that replicate all mandibular motions in static and dynamic scenarios. VA when executed in addition to other software enhances treatment planning and patient education, allowing quicker and more precise individualized diagnoses. The main objective of this study was to describe and evaluate the study outcomes in the available research on VAs, assess their needs, and evaluate their advantages and limitations in various aspects. A PubMed Central search was made of dental journals, with the identification of 135 articles out of which 30 were finally selected. The investigations conducted to evaluate the VA’s dependability provide good visualization of the quantity and location of the dynamic interactions. A precise instrument for fully analyzing occlusion in a real patient is the virtual articulator.

## Introduction and background

Digitization has become an essential aspect of modern prosthodontics, with the possibility of the majority of treatments being based on digital technology [1]. The mechanical articulator (MA) has been used in prosthodontics because it can simulate jaw movement and fix the position of the condyle. With recent advancements in technology, virtual articulator (VA) has become an important part of digital dentistry [2]. An articulator in dentistry plays a crucial role in enhancing the caliber of any dental prosthesis and improving patient satisfaction [3]. The dental articulator is described in the Glossary of Prosthodontics as “a mechanical instrument that represents the temporomandibular joints and jaws, to which maxillary and mandibular casts may be attached to simulate some or all mandibular movements” [4]. When the patient is not present, an articulator may programmed using patient information, allowing the operator to build a restoration that will be professionally and psychologically effective [5].

The concept of virtual reality is based on electronic technology, built on computers that have been connected to the near future of dental practice and dentistry. The virtual articulator is an example of virtual reality-based usage in restorative and prosthetic dentistry that considerably decreased the drawbacks of the MA through modeling of collection of the data, which allows analysis of static and dynamic occlusion as well as jaw relation of the patient. Virtual articulators are software tools that use virtual reality technology to improve healthcare outcomes [6]. Virtual articulators (VAs) are majorly classified into two following types: mathematically simulated (MS) and completely adjustable (CA). VAs are additionally useful as an educational instrument to demonstrate the patients' different treatment alternatives. Szentpetery described the first virtual articulator software in the late 1990s, while Bisler et al. from Germany launched the first VA in 2002 at the University of Greifswald [7-9].

According to Bisler et al., the clinical benefits resulting from the use of virtual articulators unlocked the opportunities for a wide range of applications in several different areas of dentistry [8]. Since then, virtual assistants have been used in computer-aided design and computer-aided manufacturing (CAD/CAM) dentistry. The first functional software of the virtual articulator is DentCam (KaVo Dental: Hamburg, Germany) which was introduced at the University of Greifswald by the aforementioned. Introducing specific patient data which includes the static and dynamic occlusal contacts during the movements of the lower jaw in DentCam software allows dentists to visualize and analyze it in a better manner. A jaw motion analyzer (JMA) was required to capture the dynamic aspects of chewing action and occlusion. This device monitors the speed of pulses of ultrasonic sound delivered using transmitters and sensors to capture mandibular motions. This extra gadget analyses mandibular motions in all three planes as follows: spatial, rotational, and translational [10]. The main objective of this study was to describe and evaluate the study outcomes in the available research on VAs, assess their needs, and evaluate their advantages and limitations in various aspects.

## Review

Search methodology

**Figure 1:**
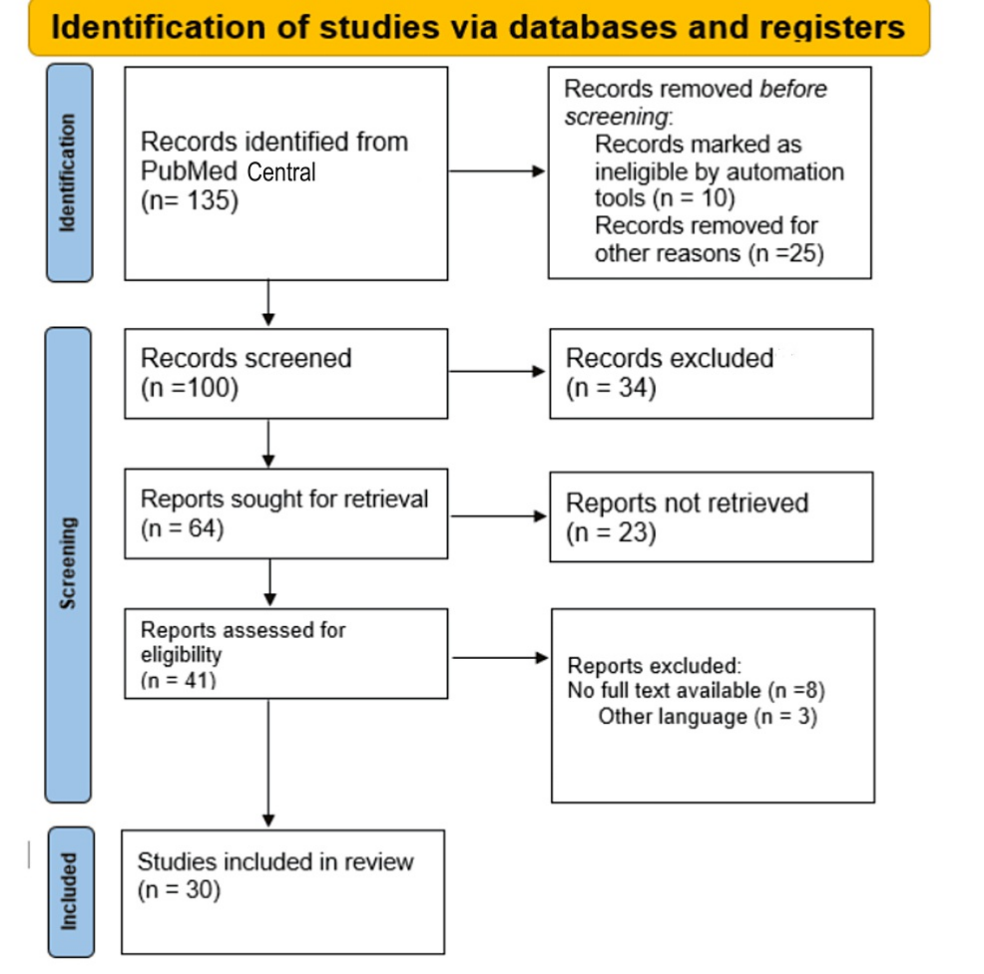
PRISMA flow diagram for literature search. PRISMA: Preferred Reporting Items for Systematic Reviews and Meta-Analyses; n: number

A comprehensive literature search strategy was implemented to identify relevant studies investigating the use of VA for dental occlusion analysis and treatment planning, compared to conventional articulators or traditional methods, resulting in improved accuracy in occlusion analysis, enhanced treatment planning, and reduced chairside time or other relevant outcomes. To achieve this, the study included both qualitative and quantitative research studies and academic articles. To find pertinent materials, a thorough search was carried out across multiple academic databases, including PubMed Central. The observation window was from August 2015 to June 2023 and the search was performed using relevant keywords and MeSH terms. The reference lists of relevant articles and review papers were manually screened to identify additional studies. To ensure the inclusion of appropriate studies, specific inclusion criteria were applied. Eligible studies needed to focus on VA, virtual reality, digitalization in dentistry, etc. Studies unrelated to digitalization, and articles lacking sufficient data were excluded. The selection process involved screening the titles and abstracts of identified articles, followed by a full-text assessment based on the inclusion and exclusion criteria. Any disagreements or uncertainties were resolved through discussions among the authors. By implementing these rigorous methodology steps, we aimed to ensure the inclusion of high-quality and relevant studies in this study, thus providing a comprehensive and reliable overview of the VAs. Figure 1 describes the selection process of articles used in this study.

Necessity of virtual articulators

The basic purpose of the VA is to enhance the designing of prostheses in dentistry along with the incorporation of analysis of kinematics into the designing procedure. The capacity of a mechanical device to imitate the differing characteristics of biological systems, i.e., the constantly changing circumstances of jaw motions and dependent features, is restricted. For example, mechanical articulators cannot imitate [5,7]. The mobility in teeth occurs whenever plaster casts are used, along with the distortion and displacement of the lower jaw amid loading circumstances.

The casts cannot accurately capture the dynamic circumstances of occlusion within the mouth. Many issues with the technological procedure and dental materials reduce the precision of reproduction. These issues can include the following: the changing shape of registration materials such as heat-sensitive waxes, inaccuracies in cast repositioning into bite imprints, articulator instability, and the proper alignment and maintenance of the MA. Because of these issues, the replication of dynamic, excessive contact appears to be less reliable. These issues can be overcome by replacing the MA with a virtual articulator. The points to be considered in mathematically stimulated (MS) and completely adjustable virtual articulators encompass various aspects. These include basic units, reproduction, drawbacks, etc. (Table 1) [11,12].

**Table 1:** Different points of consideration.

	Mathematically simulated articulators	Completely adjustable virtual articulators
Designed by	Szentpetery	Gaertner and Kordass
Basic units	Does not require extra units, it depends on mathematical simulation of articulator movements	Head stabilizer, sensor pen, transmitter, sensor for lower jaw, receiver, bite fork
Measures	Protrusion, retrusion, condyle angle, laterotrusion, Bennett angle	Calculates waves of the receiver and transmitter microphone via the triangulation method to locate the areas of patients’ mandible
Reproduction	Enables dentist to replicate the motion of an MA, which makes it a fully adjustable 3D VA	It records and reproduces the highly specific mandibular movements employing a computerized jaw recording device known as jaw motion analyzer
Drawback	The biggest drawback is that it functions as a mean value articulator and it is impossible to expect movement course of each individual	Special instruments, like a lower jaw motion-tracking system, are required. It lacks a standardized virtual format for storing lower jaw movement. As a result, this system is incompatible with a few virtual articulator software packages
Example	Szentpetery’s virtual articulators and Stratos 200 (Amherst, NY: Ivoclar Vivadent)	Stratos 300 and SAM2 (Gauting, Germany: SAM Prazisionstechnik GmbH​​​​​​​)

The completely adjustable VA uses digital tools and accessories to replicate the exact pattern of motion of the mandible. The primary indication for the CA VA type relates to complicated circumstances in which it is necessary to evaluate the occlusal plane's morphology during mandibular motions to prevent interferences with excursions. The MS type is an average value articulator; it is necessary to alter other settings to replicate mandibular movements. The primary clinical indication for the MS type pertains to situations where the prosthesis's occlusal morphology can be planned only by replicating the relationship between the arches. The primary drawback of the MS type is that it is difficult to obtain the patient's customized movements. However, the MS type is more popular because it is less costly and easier to use than the CA type [6].

Tamaki et al. conducted a study in 1977 to evaluate the replication of excursive tooth contacts with a SAM2 (Gauting, Germany: SAM Prazisionstechnik GmbH) articulator built with the aid of computerized axiography. The articulator replicated 82% of the protrusive contact of the tooth and 90% of the laterotrusive contacts of the tooth. In 66% and 81% of individuals, there were protrusive contacts and laterotrusive contacts, respectively, and the precise positions of excursive tooth interactions were replicated. Clinically, these findings imply that the articulator's capacity to recreate excursive tooth interactions is limited. When using an articulator for diagnostic and restorative dental treatments, we should keep these limitations in mind [13]. The articles included in this study very well describe the evaluation of VA, its types, concepts, advantages, and drawbacks along with its clinical implications. Digital impression made using IOS for creating functional occlusion. All 30 articles are surrounded to provide the finest knowledge to readers to enhance the dental prosthesis designing process by incorporating this virtual phase into the current technique. Table 2 depicts the summary of these 30 articles [1-3,5-11,13-32].

**Table 2:** Summary of articles included in this comprehensive review. CAD/CAM: computer-aided design and computer-aided manufacturing; 3D: three dimensional; CBCT: cone-beam computed tomography systems; IOS: intraoral scanner; VR: virtual reality SAM2 (Gauting, Germany: SAM Prazisionstechnik GmbH)

Sr. no.	Author	Year	Summary/finding
1	Bhambhani et al. [1]	2013	This article reviews multiple aspects of prosthodontics treatments where digitization has modified the traditional procedures. The article also discussed how digitalization can be useful for educational purposes, diagnosis, treatment planning, and fabrication of prostheses with its futuristic development.
2	Lepidi et al. [2]	2020	The case report described the entire outline of digital procedure from the mounting of virtual articulator to developing dynamic and static occlusion for rehabilitation of complex prosthesis.
3	Cabot [3]	1998	The article described how using the correct articulator and facebow can improve the quality of restorations.
4	Jain and Verma [5]	2016	In this article, the author described the history of evaluation of the articulators, over the past 19 years from 1951 to 1970.
5	Koralakunt and Aljanakh [6]	2014	The goal of the article is to present the strategy and ideas for replacing the mechanical articulator with a virtual articulator. And the author has also given a brief about the virtual reality haptic system.
6	Kordass et al [7]	2002	The article discussed the developments of virtual reality, its concepts, its advantages, and clinical implications.
7	Bisler et al. [8]	2002	The article discussed the potential of virtual reality for implant planning and design when paired with CAD/CAM technology which leads to increased precision and a reduced treatment time.
8	Gärtner and Kordass [9]	2003	The article examined the virtual articulator's precision, dependability, and repeatability when used in functional diagnostics. Additionally investigated is its potential for planning and modeling orthodontic treatment.
9	Lepidi et al. [10]	2021	This article reviewed the history of virtual articulators with newly created methods and procedures for virtual mounting that make use of modern technology and knowledge.
10	Özdemir et al. [11]	2021	This article examines the specifications for occlusal recordings and virtual articulators, weighing the benefits and drawbacks from a number of angles.
11	Tamaki et al. [13]	1997	The study's goal was to evaluate, using computerized axiography, how well the SAM2 articulator setup reproduced excursive tooth interactions. It was discovered that the articulator's capacity to do so was limited.
12	Ruge and Kordass [14]	2008	The study on dynamic occlusion is carried out on Zebris company and D-Isny module system on 3D virtual articulator.
13	Schoenbaum [15]	2012	This article was published in the year 2012 and provides the all-latest update regarding virtual dentistry.
14	Kim et al. [16]	2021	The article describes the technique of using CBCT data to duplicate the plane of occlusal in relation to digital scan data, which is transferred to a mechanical articulator.
15	Lepidi et al. [17]	2019	The article describes a complete digital method that uses intraoral scans and a virtual articulator to transfer the location of the maxillary dentition along with CBCT files
16	Solaberrieta et al. [18]	2013	The article describes the position of directly collected digital castings that can be mapped to a virtual articulator using the digital approach, which leads to significant time savings and a more accurate cast position determination.
17	Maestre-Ferrín et al. [19]	2012	This article reviewed the creation, operation, and use of virtual articulators that have been published in the literature.
18	Úry et al. [20]	2020	The goal of the clinical investigation was to determine how accurate the indirect digital workflow-based virtual dental environment was and came to the conclusion that virtual occlusal analysis in clinical practice can be effectively performed using the virtual dental space produced by the indirect digital method.
19	Abad-Coronel et al. [21]	2019	This is a review article that provides knowledge and information about digital impressions intraorally. It described the different IOS systems used along with their advantages and disadvantages over conventional impressions.
20	Ender et al. [22]	2016	This in vivo study looked into the precision of traditional and digital techniques of full-arch impressions.
21	Keul and Güth [23]	2020	This article compared the full-arch digital impressions against traditional impressions in vivo and in vitro manner
22	Hsu and Mehl [24]	2019	The author studied the impact of changing pin openings and condylar settings on virtual articulators’ accuracy and trueness in comparison to mechanical articulators
23	Ender et al. [25]	2013	The study is to evaluate intraoral digital impressions' accuracy and contrast them with traditionally obtained impressions.
24	Solaberrieta et al. [26]	2016	The article aims to describe, imitate, and analyze mandibular motions of the human jaw through the building of a virtual articulator. Its primary objective is to enhance dental prosthesis design by incorporating kinematic analysis into the design process.
25	Song and Baek [27]	2009	The author compared the precision of traditional manual and three-dimensional virtual techniques for intermediate wafer fabrication and model surgery.
26	Abu et al. [28]	2021	This paper aims to provide an overview of dental articulators, including their indications, benefits, and drawbacks. There is a useful resource available to help choose the best articulator for specific clinical circumstances.
27	Solaberrieta et al. [29]	2009	This article determined the quantity, requirements, and virtual occlusal record dimension which finds the lower arch cast's Three-dimensional spatial location in relation to the upper arch cast on a virtual articulator.
28	Kalpana et al. [30]	2018	This article's goal is to illustrate the steps and advantages of virtual articulators over conventional ones.
29	Ikawa et al. [31]	2011	The author described a method for designing a functional occlusal surface by simulating lateral excursions using the VR articulator.
30	Solaberrieta et al. [32]	2013	This article presents research that aims to create a digital facebow that can be used to find maxillary casts on a virtual dental articulator. Its primary objective is to enhance the dental prosthesis design process by incorporating this virtual phase into the current technique.

Recent developments of virtual articulators

The following three key items are required for the building of a three-dimensional (3D) virtual articulator system: a 3D scanner as an input device, the software of 3D VA for prosthesis and collision detection, and an output device - stereoscopic inkjet technology "rapid prototyping system." The fundamental advantage of a 3D virtual articulator technology is that it can examine functional masticatory motions, including the use of force sites to reach out and the rate of contact in terms of time and in addition to mandibular movements [14,15].

Discussion

The virtual articulator is a computer-programmed method for analyzing static and dynamic occlusion interactions. Its principal applications include individual diagnostics and MA simulation [4]. Various writers have examined virtual articulators (VAs), and the approaches utilized for constructing computer-generated images of the jaw represented in a VA have undergone significant advancements and modifications [16-19]. The definition of the processes of virtual articulator assembly is direct or indirect depending on whether analog stages have been included during data collecting and transmission operations [20]. VA assembly consists of four major steps as follows: impression of the arches, occlusal registration, virtual facebow, and virtual mounting.

The intraoral scanner (IOS) is responsible for the initial part of the digital workflow, which is picture acquisition. These devices are gaining popularity as an alternative to traditional impressions created using trays and elastomeric materials [21]. Acquisition of data from the upper arches using an intraoral scanner has been proven to be more accurate than analog approaches [22,23]. Additionally, direct digital impressions outperform the existing reference standard for patient-investigating models, which are typically mounted in a mechanical articulator. The digital articulator's dynamic motions were proved to be as precise and accurate as the conventional articulator's [24]. In routine practice for both upper and lower arches, the digital impressions have demonstrated greater accuracy than indirect scanning of stone casts or impressions with a desktop laboratory scanner. Physical models of 3D can also be printed with digital impressions. Some guidelines have been used to decrease errors during the immediate capture for both the arch by IOS, firstly the scanning of occlusal surfaces of teeth should be done, with some acquisitions as feasible to have reduced overlapping which is more ideal [25,26].

The most realistic occlusal surface replication may be obtained by employing either a fully adjustable articulator that replicates lower jaw movements with utmost accuracy or VA with CAD/CAM systems [27]. The digital articulator combined with CAD/CAM system has significant potential in treatment planning with dental implants since it allows more accuracy and reduces the time of implant therapy [28]. In orthognathic surgery, the VA was compared with the conventional articulator to create optimal maxillary position and to prepare surgical splints, with the conclusion that the digital method is more exact than the conventional one [28]. Virtual articulators are quite dependable. Multiple other studies suggested the quantity and position of variable occlusal surfaces captured by a virtual articulator, these were found to be identical to those obtained by mechanical one [29].

The operation of the virtual articulator relies on the development of an image of the mandible's motions according to the input data, which estimates the points of occlusion, which is then displayed on the screen of the computer via a particular type of coding [6]. Mandibular movements cannot be reproduced by the use of semi-adjustable mechanical articulators, with relation to time periods. This can be overcome by swapping MA by digitally replicating to VA. The virtual articulator can represent and measure the impacts of soft tissue resilience on a time-dependent basis during masticatory actions. As a result, it can demonstrate the occlusion's real-time dynamics. The goal of the virtual articulator is to serve as an analytical tool for the intricate static and dynamic occlusal relations. The virtual articulator's primary objective is to enhance dental prosthesis design by including kinematic analysis in the designing process. Similar to other contemporary methods and technologies, utilizing the virtual articulator necessitates a comprehensive comprehension of its principles and operational mechanisms [30,31]. The main results obtained from the 30 selected articles concluded that VA has a greater convenience and accuracy over traditional articulators though it has its limitations [1-32].

Programming the virtual articulators

Koralakunte and Aljanakh described the programming of VA. The functioning method of virtual articulators is based on the input data, the VA's fundamental system develops an animation of the mandibular movements and determines the points of occlusion, which are then shown on the computer screen for which the scanning is done [6]. It can be done in two ways direct scanning and indirect scanning. Direct scanning is done directly into patient's mouth using IOS and indirect scanning is done outside using cast models. Once the scanning is done the motion model is assembled and checked for virtual simulation design. If there is any problem detected it should be redesigned and rechecked at this stage.

The designing method of VA is initially similar to the functioning method of VA. First, the 3D scanning of data is done and then this is loaded on the virtual jaw models. The motion data of the temporomandibular joints for the specific patient is recorded either by using JMA tool or motion parameters. The JMA tool, which has a reference point positioned on the mandible from which patient-specific jaw motion data is acquired and occlusion verification is carried out visualizing on 3D VA, is used to input jaw motion data into the motion analyzer tool. The motion parameters are opted for when the JMA tool is not available, different jaw motions can be defined via parameters as used within the mechanical articulators, such as protrusion, retrusion, laterotrusion, and opening and closing movement of mandible. Once the loading of motion-defining parameters is fixed, the constrained jaw movements are simulated and the occlusion verification is done by visualizing on 3D VA [6].

Advantages 

Advantages include that it enables the highest level of interaction among the dentist, dental technician, and patient. It works in conjunction with computer-aided design and computer-aided manufacturing systems to develop the occlusal surface, also examines both static and dynamic occlusion, analyses gnathic and joint disorders, eliminates manufacturing problems, and can visualize specific regions in 3D. It simplifies operations for the dental technician along with the dentist and it is more time efficient and can even imitate the patient. It supports in patient education and rehabilitation as well [11].

Disadvantages

The main disadvantage of these virtual articulators is that the casts have to be mounted with gypsum onto the physical articulator using a physical facebow, and then, they must be scanned in this position. Apart from a physical articulator, this is a tedious step that requires a long time [32]. It has certain limitations including the cost, since it involves software, digital scanners, and sensors, several types of VA models that imitate conventional ones based on the individual’s needs, the expertise of computer-aided design and computer-aided manufacturing systems, manual articulators, design and simulation of virtual articulators, and technical abilities in interpreting data acquired from of sensors, scanner, minor changes, integrating motion parameter, and so on [11].

## Conclusions

The advent of augmented reality innovation has enabled dental practitioners to successfully diagnose and arrange treatment utilizing a virtual articulator in daily clinical practice. The VA is an accurate piece of software that operates in combination with CAD/CAM systems to replace MA and thereby prevent faults. In dynamic simulations, these systems can be improved by utilizing four-dimensional (4D) technology in the upcoming years. These are the reasons why virtual reality has gained popularity and transformed dental practices. The virtual articulators represent a promising technological advancement in which patients will be more comfortably treated with greater convenience and precision.
